# Survival and glycemic control in patients with co-existing squamous cell carcinoma and diabetes mellitus

**DOI:** 10.2144/fsoa-2020-0150

**Published:** 2021-02-10

**Authors:** Sophia A Ederaine, Johanny Lopez Dominguez, Jamison A Harvey, Aaron R Mangold, Curtiss B Cook, Heidi Kosiorek, Matthew Buras, Kyle Coppola, Nina J Karlin

**Affiliations:** 1 Mayo Clinic Alix School of Medicine, Scottsdale, AZ 85259, USA; 2 Department of Dermatology, Mayo Clinic, Scottsdale, AZ 85259, USA; 3 Division of Endocrinology, Mayo Clinic, Scottsdale, AZ 85259, USA; 4 Department of Health Sciences Research, Mayo Clinic, Scottsdale, AZ 85259, USA; 5 Department of Cancer Quality Program, Mayo Clinic, Scottsdale, AZ 85259, USA; 6 Department of Information Technology, Mayo Clinic, Phoenix, AZ 85054, USA; 7 Division of Hematology & Medical Oncology, Mayo Clinic Cancer Center, Phoenix, AZ 85054, USA

**Keywords:** cancer, diabetes, endocrinology, glycemic control, outcomes research, squamous cell carcinoma

## Abstract

**Aim:**

This study examined the impact of diabetes mellitus (DM) on survival in squamous cell carcinoma (SCC) patients, and the impact of SCC on glycemic control.

**Materials & methods:**

Patients with newly diagnosed SCC with and without DM were matched 1:1 (2007–2017). Overall survival and recurrence-free survival were estimated using the Kaplan–Meier method. Hemoglobin A_1c_ (HbA_1c_) and glucose level during the year following cancer diagnosis were compared using mixed models.

**Results:**

HbA_1c_ decreased over time in DM patients (p = 0.04). The 5-year overall survival was 61% in DM patients, compared with 78% in patients without DM (p = 0.004).

**Conclusion:**

The presence of co-existing DM adversely impacted survival in patients with SCC. SCC did not affect glycemic control.

## Lay abstract

The objective of this study was to identify the effect of diabetes mellitus (DM) on survival of patients with squamous cell carcinoma (SCC) and to determine whether SCC and its treatment affected glycemic control. We used an institutional cancer registry to identify 190 patients with SCC and grouped them by the presence (n = 95) or absence (n = 95) of DM. Patients were matched by age and year of SCC diagnosis. For individuals with SCC, DM did decrease survival rates, and the diagnosis of SCC did not affect glycemic control.

Squamous cell carcinoma (SCC) is one of the most common cancers worldwide [1]. SCC is characterized by high morbidity and mortality due to its invasive nature, often spreading to neighboring tissues and metastasizing to distant sites [2,3]. Diabetes mellitus (DM) is one of the most common chronic conditions in the USA [4]. This high prevalence is encountered in cancer patients as 10% have a co-existing DM diagnosis [5]. Previous studies have alluded that a relationship between diabetes and certain cancers may be causal and that diabetes can decrease the survival in patients with cancer [6–9].

Recent studies have found an increased risk of developing SCC in patients with DM [10,11]. A retrospective study showed the incidence of developing overall skin cancer and nonmelanoma skin cancer to be 1.29-times higher in DM patients above the age of 60 years [10]. Another study suggested that diabetes may be a risk factor for oral squamous premalignancies and tumors. Oral manifestation of diabetes, which includes inflammatory process and atrophic lesions have been proposed as being possible precursors to malignant transformation [12].

Previous studies from our institution have evaluated the relationship between patients with DM and several malignancies including melanoma, pancreatic cancer, lung cancer, breast cancer, prostate cancer, gastroesophageal cancer, colorectal cancer and lymphoma [13–20]. Only gastroesophageal cancer has shown a decrease in survival in patients with DM compared with those without [18]. This study aims to identify the effect of DM on SCC survival and whether SCC and its treatment, affect glycemic control among patients with DM.

## Materials & methods

### Case selection

After institutional review board approval, patients were identified from the institutional cancer registry and a retrospective review of electronic medical records of patients with newly diagnosed SCC from 1 January 2007 to 31 December 2017 was performed. Age at SCC diagnosis, diagnosis date, race/ethnicity and grade or stage of tumor was obtained. SCC was defined as head and neck cutaneous SCC and oropharyngeal SCC.

All patient data were crossreferenced against a list of all patients seen during the study period who also had a known diagnosis of DM. Type 1 and Type 2 DM were included. We excluded patients who received full or partial treatment at another institution or who had another primary cancer. From this dataset, patients with SCC and DM were matched to patients with SCC and without DM at a 1:1 ratio by using a Greedy algorithm [21,22]. Variables included in the matching algorithm were age, sex and year of SCC diagnosis. Year of SCC diagnosis was used as a matching variable to ensure a similar duration of follow-up for patients with and without DM.

Data was obtained for type of SCC treatment (surgery, chemotherapy, radiotherapy, targeted therapy), glucose values, date of DM diagnosis, medications for DM treatment (diet, oral, oral + insulin, insulin and other), complications of DM, hemoglobin A1c of the diabetic patients.

### Statistical analysis

The statistical analyses conducted were similar to those used for our previous studies [13–20]. Patients with DM (cases) and without DM (controls) were compared on the basis of patient demographic and clinical variables. Categorical variables were compared using the McNemar test or the Bowker test while continuous variables were compared using paired *t*-tests. HbA_1c_ levels during the first year after SCC diagnosis were analyzed with a linear mixed model in the DM group only (HbA_1c_ values were not available for the majority of patients without DM). Time (days) was considered a fixed effect, and an individual-specific random effect was included. A similar approach was used for modeling glucose values during the first year. Fixed effects included days, case or control designation, an interaction term (days × case–control designation) and patient-specific and matched pair-specific random effects. Optimal glycemic control was defined as a mean glucose value less than 126 mg/dl during the year after cancer diagnosis.

Overall survival (OS) was defined as the time from SCC diagnosis until death from any cause. For OS, patients were considered censored at the last known follow-up date if death was not documented in the health records. Two-year OS was estimated with the Kaplan–Meier method and compared between groups by using the log-rank test. Cox proportional hazards regression was used to assess for effect of DM on OS and included matched pairs as the strata variable. Sample size was based on the number of available cases from 2007 to 2017; it provided 80% power to detect a difference in hazard ratio (HR) of 1.9 or greater for OS. The p-values < 0.05 were considered statistically significant. SAS version 9.4 (SAS Institute Inc., NC, USA) was used for analysis. Data for continuous variables were reported as mean standard deviation (SD) and categorical variables as percentage.

## Results

### Patient characteristics

We analyzed 95 matched pairs (Table 1). Mean (SD) age at diagnosis was 66.1 (10.22) years, 83.2% were male (n = 158), 181 (95.3%) were Caucasian and 60.3% had stage IV disease. There was a significant difference in ethnicity between patients that had DM and patients without DM. 9.6% of patients with DM identified as Hispanic, compared with 1.1% of patients without DM (p = 0.010). Patients with DM had a higher BMI (mean [SD], 29.7 [6.94] vs 28.0 [4.17] kg/m^2^; p = 0.041). More patients without DM had alcohol consumption at time of diagnosis compared with those with DM (76.8 vs 51.6%; p < 0.001). More patients with DM were former smokers compared with those without DM (64.2 vs 40%), but more patients without DM were current smokers compared with those with DM (22.1 vs 7.4%; p = 0.008).

**Table 1.** Characteristics of patients with squamous cell carcinoma.

	Diabetes			
	No (n = 95)	Yes (n = 95)	Total (n = 190)	p-value
Current age (years), mean (SD)	70.8 (9.88)	70.1 (10.63)	70.5 (10.24)	0.0521
Age at diagnosis (years), mean (SD)	66.0 (10.18)	66.1 (10.32)	66.1 (10.22)	Matched
Male sex, n (%)	79 (83.2%)	79 (83.2%)	158 (83.2%)	Matched
White race, n (%)	92 (96.8%)	89 (93.7%)	181 (95.3%)	0.2942
Ethnicity				0.0102
Hispanic	1 (1.1%)	9 (9.6%)	10 (5.3%)	
Non-Hispanic	84 (88.4%)	69 (73.4%)	153 (81.0%)	
Unknown	10 (10.5%)	16 (17.0%)	26 (13.8%)	
Missing	0	1	1	
Anatomic location, n (%)				0.8482
Lip	6 (6.3%)	6 (6.3%)	12 (6.3%)	
Oral cavity	20 (21.1%)	28 (29.5%)	48 (25.3%)	
Oropharynx	63 (66.3%)	54 (56.8%)	117 (61.6%)	
Hypopharynx	2 (2.1%)	4 (4.2%)	6 (3.2%)	
Nasopharynx	3 (3.2%)	2 (2.1%)	5 (2.6%)	
Salivary gland tumor	1 (1.1%)	0 (0.0%)	1 (0.5%)	
Other	0 (0.0%)	1 (1.1%)	1 (0.5%)	
Surgery, n (%)				0.3282
No	12 (12.8%)	17 (17.9%)	29 (15.3%)	
Yes	82 (87.2%)	78 (82.1%)	160 (84.7%)	
Tumor stage, n (%)				0.8032
I	17 (18.7%)	12 (13.6%)	29 (16.2%)	
II	9 (9.9%)	7 (8.0%)	16 (8.9%)	
III	15 (16.5%)	11 (12.5%)	26 (14.5%)	
IV	50 (54.9%)	58 (65.9%)	108 (60.3%)	
Missing	4	7	11	
BMI, mean (SD), kg/m^2^	28.0 (4.17)	29.7 (6.94)	28.8 (5.75)	0.0411
Married at time of cancer diagnosis, n (%)	71 (74.7%)	64 (67.4%)	135 (71.1%)	0.9042
Unknown	2 (2.1%)	2 (2.1%)	4 (2.1%)	
Any alcohol at time of cancer diagnosis, n (%)				<0.001^†^
Yes	73 (76.8%)	49 (51.6%)	122 (64.2%)	
No	22 (23.2%)	44 (46.3%)	66 (34.7%)	
Unknown	0 (0.0%)	2 (2.1%)	2 (1.1%)	
Smoking status at time of cancer diagnosis, n (%)				0.0082
Never	36 (37.9%)	27 (28.4%)	63 (33.2%)	
Former	38 (40.0%)	61 (64.2%)	99 (52.1%)	
Current	21 (22.1%)	7 (7.4%)	28 (14.7%)	
Eastern Cooperative Oncology Group performance status at time of cancer diagnosis, no (%)				0.4802
0	41 (43.2%)	29 (32.2%)	70 (37.8%)	
1	15 (15.8%)	16 (17.8%)	31 (16.8%)	
2	1 (1.1%)	2 (2.2%)	3 (1.6%)	
3	1 (1.1%)	3 (3.3%)	4 (2.2%)	
4	1 (1.1%)	0 (0.0%)	1 (0.5%)	
Unknown	36 (37.9%)	40 (44.4%)	76 (41.1%)	
Missing	0	5	5	
Use of steroids, n (%)				0.1502
Yes	24 (25.5%)	32 (35.6%)	56 (30.4%)	
No	70 (74.5%)	58 (64.4%)	128 (69.6%)	
Missing	1	5	6	

† McNemar’s or Bowker’s test for symmetry.

SD: Standard deviation.

### DM & SCC treatment characteristics

For the majority of patients (96.6%), DM diagnosis preceded SCC diagnosis (Table 2). With most of the patients receiving oral therapy (n = 50 [57.5%]) for DM at the time of their SCC diagnosis. There were 16 patients (18.6%) who changed their DM therapy within 1 year after the squamous carcinoma cancer diagnosis; seven patients (43.8%) used insulin within 1 year after the cancer diagnosis. Complications from DM were documented for four patients (4.9%) within 1 year after SCC diagnosis. Corticosteroids were taken by 25.5% of patients without DM and 35.6% of patients with DM (p = 0.150). There were no significant differences in cancer therapies (surgery, chemotherapy, radiotherapy and targeted therapy) noted between patients with and without DM.

**Table 2.** Diabetes mellitus treatment for patients with squamous cell carcinoma.

Diabetes diagnosis preceded SCC diagnosis, n (%)	84 (96.6%)
Diabetes therapy, n (%)	
Diet	11 (12.6%)
Oral	50 (57.5%)
Insulin	16 (18.4%)
Oral + Insulin	9 (10.3%)
Other	1 (1.1%)
Missing	8

SCC: Squamous cell carcinoma.

### SCC effect on DM & metabolic control

**Figure 1.**
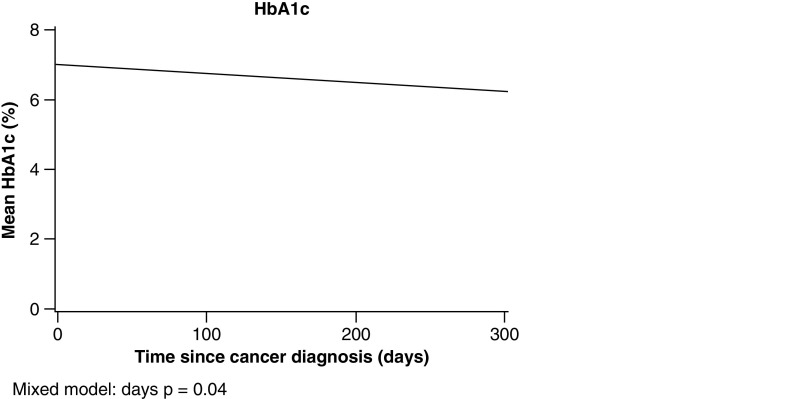
Estimated mean hemoglobin A1c value during year after squamous cell carcinoma diagnosis in patients with diabetes mellitus.

The HbA_1c_ data, measured within 1 year after the SCC diagnosis were available for 70 patients (73.6%) with DM. Mean (SD) HbA_1c_ was 6.7% (1.2%) and 31.43% of these patients had at least one HbA_1c_ measurement of greater than or equal to 7.0% within 1 year of the SCC. Mean HbA_1c_ among DM patients decreased significantly over time (p = 0.04) (Figure 1). For glucose, DM group status was significant (p < 0.001) as DM patients had higher glucose overall compared with non-DM. In mixed model analyses, HbA_1c_ decreased over time in DM patients (p = 0.04).

**Figure 2.**
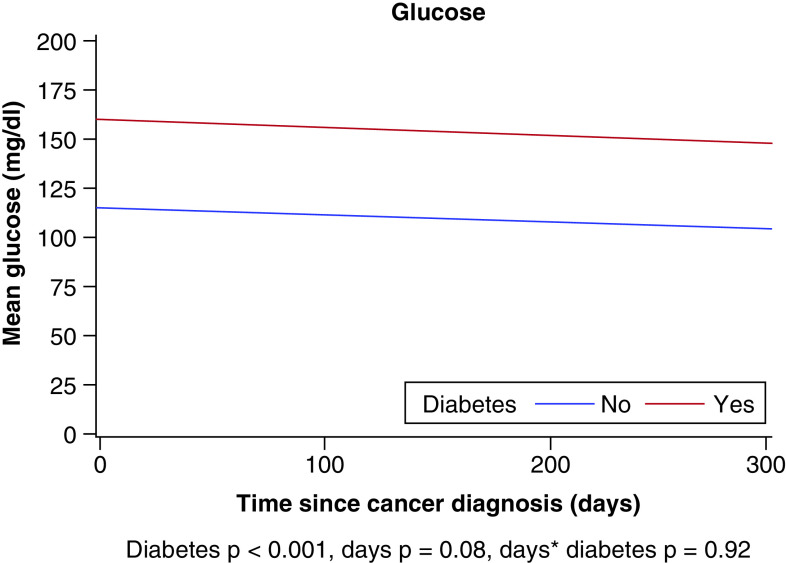
Estimated mean glucose value during first year after squamous cell carcinoma diagnosis.

Mean glucose values during the year after SCC were significantly different between patients with DM (153.4 mg/dl) and patients without DM (109.8 mg/dl; p < 0.001) (Figure 2). Neither group had a decline in glucose values during the 1 year after cancer diagnosis; we did not observe any significant interaction effect (p = 0.92) or time effect (p = 0.08).

### DM effect on SCC survival

**Figure 3.**
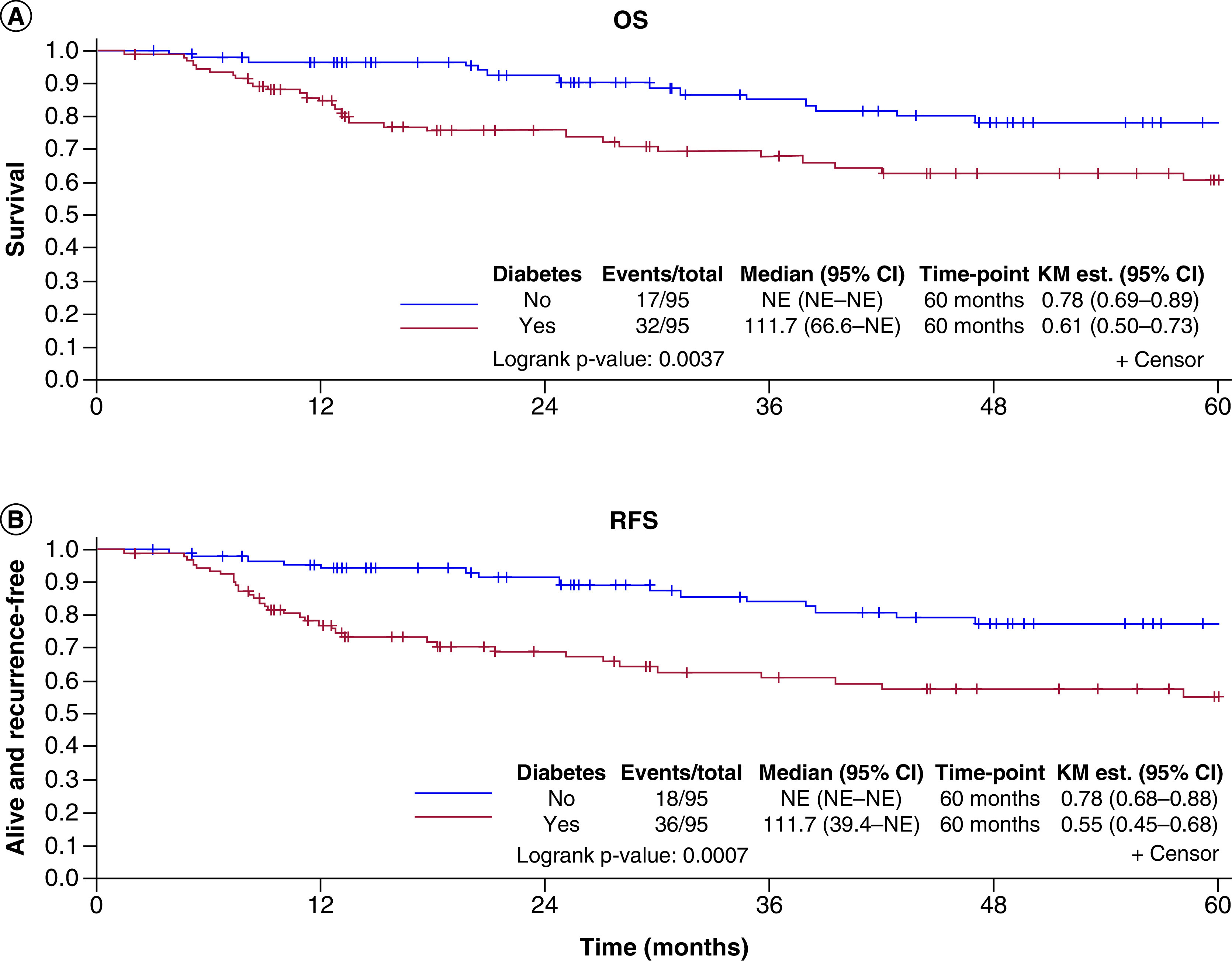
Overall survival and recurrence free survival. KM: Kaplan-Meier; NE: Not estimated; OS: Overall survival; RFS: Recurrence-free survival.

In patients with DM, the 5-year OS was 61% (95% CI: 0.50–0.73), compared with 78% (95% CI: 0.69–0.89) in patients without DM (p = 0.004) (Figure 3). The HR for matched pairs was 2.60 (95% CI: 1.25–5.39; p = 0.01). The HR remained significant at 2.90 (95% CI: 1.11–7.57; p = 0.03) for OS when adjusting for smoking status and BMI. The 5-year RFS was 55% for patients with DM compared with 78% for patients without DM (p < 0.001) (HR: 3.33; 95% CI: 1.58−7.02; p = 0.001) (Figure 3). The HR remained significant at (HR: 3.92; 95% CI: 1.54–9.90; p = 0.004) for RFS when adjusting for smoking and BMI.

## Discussion

This study shows that in our cohort, patients with SCC and DM have a lower 5-year OS than those without DM. In addition, glycemic control did not deteriorate among the DM patients over the 1-year period after cancer diagnosis. The results of this study are similar to those found in prior studies that only focused on oral cavity cancer, with lower OS in DM patients compared with their non-DM cohort [23–25]. They found the 6-year OS for patients with DM was 56.8% compared with 68.7% for non-DM patients [23].

In comparison, another study looked at OS in patients with head and neck SCC (SCC of the oral cavity, oropharynx, larynx and hypopharynx). They found that compared with patients with DM, there was no statistically significant difference in the 5-year OS to non-DM patients (64 vs 67%) [26]. The differences in our results may be due to the fact that SCC for us was defined as head and neck cutaneous SCC and oropharyngeal SCC.

In this current study, we used a case–control method to learn more about the effect of diabetes on SCC survival in the USA, within a specific US healthcare system. Our findings found statistical significance compared with the other multiple case–control studies done at our institution. The authors had previously investigated the effect of solid tumors – breast, lung, prostate, colorectal, pancreatic, gastric, esophageal, lymphoma and melanoma and DM on patient outcome measures [13–20]. In all these case–control studies, DM did not affect survival of patients. Like previous studies from our institution on solid organ tumors, this study also showed that SCC did not affect glycemic control after a 1-year follow-up.

The pathogenesis of DM could influence the decreased survival of SCC. DM and SCC share common risk factors, such as hyperglycemia, hyperinsulinemia, insulin resistance, chronic inflammation and immune system dysfunction [27]. Tobacco smoking and alcohol which are two of the etiologic agents of SCC, particularly head and neck SCC and oropharyngeal SCC are also a risk factor in diabetes [28–30]. Tobacco is one of the major risk factors that may have been the confounding variable causing decreased survival in DM patients with SCC, compared with their non-DM counterparts. However, in our study, when adjusting for smoking and BMI, the differences seen in OS and RFS between DM and non-DM patients remained significant on multivariate analyses.

From our study, diabetic patients did not have worsening of their disease because SCC did not affect glycemic control. However, how DM therapy affects SCC has been explored in other reports. Some studies have explored the impact that metformin therapy has on SCC treatment and the data are mixed. Some suggests metformin reduces progression of SCC and may illicit antitumorigenic immune response [31–33]. While other data does not show association between metformin and oncologic outcomes [34].

There are limitations that should be considered when interpreting the results of our study. Most patients in the study were white and thus these results might be less applicable to patients of other ethnic and racial backgrounds. Additionally, we were not able to determine disease specific survival. Additionally, the treatments associated with a high stage SCC may affect glycemic control (corticosteroids and chemotherapies) and this is a possible confounding factor.

## Conclusion

This research further increases our understanding of the connection between diabetes and SCCs. There have been few studies to investigate how glycemic control affects different kinds of SCCs, including head and neck squamous cell carcinoma (HNSCC) and oropharyngeal SCCs. It is also one of the few published US-based study that evaluated the effect of diabetes on SCC survival. In the analysis, diabetes decreased SCC survival, but did not affect glycemic control.

## Future perspective

With the findings of this study, providers should be aware of the negative effects of DM in the outcomes of patients with SCC. Providers can be reassured that SCC does not negatively affect glycemic control among patients with DM. Future studies are needed to explore the effect that the findings of this study has in terms of treatment of SCC in patients with DM. It would also be interesting to review the long-term effect on glycemic control after cancer diagnosis.

**Summary points**

- Squamous cell carcinoma (SCC) is one of the most common cancers worldwide. Additionally, diabetes mellitus (DM) is a high prevalent comorbidity and the amount of patients with DM is expected to significantly increase. Recent studies have found an increased risk of developing SCC in patients with DM. There is still a limited understanding of the effects that DM and its treatment have on SCC outcomes.
- This was a case–control study of 95 matched pairs of SCC patients with and without DM. Mean age was 66.1 years, 83.2% were male, 95.3% were Caucasian and 60.3% had Stage IV disease.
- Mean glucose values during the year after SCC were significantly different between patients with DM (153.4 mg/dl) and patients without DM (109.8 mg/dl; p < 0.001) (Figure 2). Neither group had a decline in glucose values during the 1 year after cancer diagnosis, but patients with DM had a higher BMI (mean [SD], 29.7 [6.94] vs 28.0 [4.17] kg/m^2^; p = 0.041).
- In patients with DM, the 5-year overall survival (OS) was 61% (95% CI 0.50–0.73), compared with 78% (95% CI 0.69–0.89) in patients without DM (p = 0.004). The hazard ratio (HR) for matched pairs was 2.60 (95% CI: 1.25–5.39; p = 0.01). The 5-year RFS was 55% for patients with DM compared with 78% for patients without DM (p < 0.001) (HR: 3.33; 95% CI: 1.58−7.02; p = 0.001) (Figure 3).
- Providers should be aware of the negative effects of DM in the outcomes of patients with SCC. Future studies are needed to explore the effect that the findings of this study has in terms of treatment of SCC in patients with DM.
